# Supplementary material for: Increased Collagen I/Collagen III Ratio Is Associated with Hemorrhage in Brain Arteriovenous Malformations in Human and Mouse
Source: Cells. 2024 Jan 1;13(1):92. doi: 10.3390/cells13010092 (PMC10778117; doi:10.3390/cells13010092)
Supplement: Supplementary file 1 [file cells-13-00092-s001.zip › Supplementary Figure and table.pdf]

Increased Collagen I/Collagen III Ratio Is Associated with Hemorrhage in Brain Arteriovenous Malformations in Human and Mouse

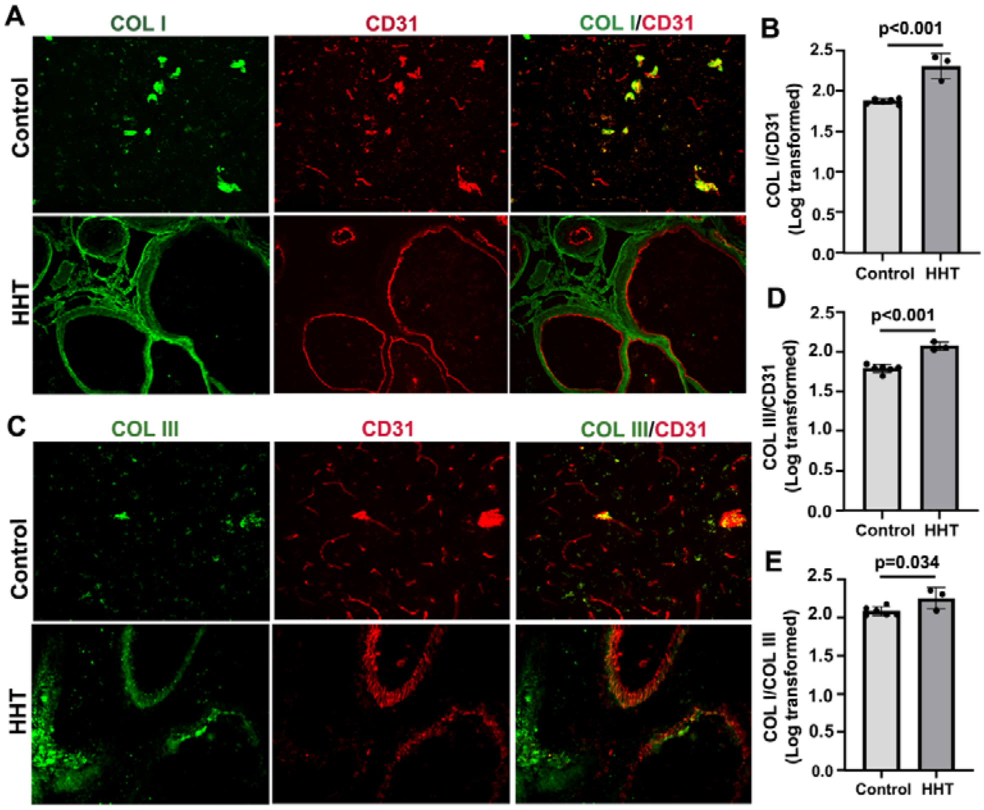

Supplementary Figure S1: COL I and COL III levels and COL I/ COL III ratio are higher in human HHT bAVMs compared to control. **A & C.** Representative images of COL I (green) and COL III (green). stained sections. ECs (red) were stained by an anti-CD31 antibody. **Scale bar**=50 μm. **B & D.** Quantifications of COL I and COL III levels. **E.** Quantification of COL I/COL III ratio. N=6 for control, N=3 for HHT bAVMs.

Supplemental Table S1. Demographic information of individuals in the Sample group

study_id	sex	Cohort	Presentation	AGE	race	ethnicity
112365	male	AVM	Ruptured	51	Asian	No
112411	male	AVM	Unruptured	61	White	No
112412	female	AVM	Ruptured	50	More than one race	Yes
112430	male	AVM	Unruptured	59	White	No
112442	male	AVM	Ruptured	61	White	No
112496	female	AVM	Ruptured	52	White	No
112655	female	AVM	Unruptured	57	White	No
112678	male	AVM	Ruptured	62	White	Yes
112761	male	AVM	Unruptured	53	White	No

113103	female	AVM	Unruptured	51	White	No
113105	female	AVM	Unruptured	46	White	Yes
113186	female	AVM	Ruptured	46	White	No
900000	Male	Epilepsy	Epilepsy	44	White	Unknown
900015	Male	Epilepsy	Epilepsy	50	White	No
900015	Male	Epilepsy	Epilepsy	50	White	No
900032	Female	Epilepsy	Epilepsy	46	White	Unknown
900037	Female	Epilepsy	Epilepsy	65	White	No
900073	Female	Epilepsy	Epilepsy	55	White	No
112488	Male	HHT AVM	Unruptured	69.1	White	No
112823	Female	HHT AVM	Unruptured	48.8	White	Yes
113098	Male	HHT AVM	Unruptured	9.1	More than one race	Yes
